# The EBMT activity survey on hematopoietic-cell transplantation and cellular therapy 2018: CAR-T’s come into focus

**DOI:** 10.1038/s41409-020-0826-4

**Published:** 2020-02-17

**Authors:** Jakob R. Passweg, Helen Baldomero, Christian Chabannon, Grzegorz W. Basak, Selim Corbacioglu, Rafael Duarte, Harry Dolstra, Arjan C. Lankester, Mohamad Mohty, Silvia Montoto, Régis Peffault de Latour, John A. Snowden, Jan Styczynski, Ibrahim Yakoub-Agha, Nicolaus Kröger, for the European Society for Blood and Marrow Transplantation (EBMT)

**Affiliations:** 1 EBMT Activity Survey Office, Hematology, Department of Medicine, University Hospital, Basel, Switzerland; 2 Institut Paoli Calmettes & Inserm CBT-1409, Centres d’Investigations Cliniques en Biothérapies, Marseille, France; 3 Department of Hematology, Oncology and Internal Medicine, Medical University of Warsaw, Warsaw, Poland; 4 Pediatric Hematology, Oncology and Stem Cell Transplantation Department, Children’s Hospital, Regensburg, Germany; 5 Servicio de Hematologia y Hemoterapia, Hospital Universitario Puerta de Hierro, Madrid, Spain; 6 Laboratory of Hematology, Department of Laboratory Medicine, Radboud University Medical Center, Nijmegen, The Netherlands; 7 Willem-Alexander Children’s Hospital, Department of Pediatrics, Leiden University Medical Centre Leiden, Leiden, The Netherlands; 8 Department of Hematology, Hospital Saint Antoine, Paris, France; 9 St. Bartholomew’s Hospital, Barts Health NHS Trust, London, UK; 10 BMT Unit, Department of Hematology, Hospital St. Louis, Paris, France; 11 Department of Haematology, Sheffield Teaching Hospitals NHS Foundation Trust, Sheffield, UK; 12 Department of Pediatric Hematology and Oncology, University Hospital, Collegium Medicum UMK, Bydgoszcz, Poland; 13 CHU de Lille, LIRIC, INSERM U995, Université de Lille, 59000 Lille, France; 14 Department of Stem Cell Transplantation, University Hospital Eppendorf, Hamburg, Germany

**Keywords:** Haematological cancer, Leukaemia

## Abstract

Hematopoietic-cell transplantation (HCT) is widely used for acquired and congenital disorders of the hematopoietic system. Number of transplants performed in Europe and associated countries continues to rise with 47,468 HCT in 42,901 patients [19,630 allogeneic (41%) and 27,838 autologous (59%)] reported by 701 centers in 50 countries in 2018. Main indications were myeloid malignancies 10,679 (25%; 97% allogeneic), lymphoid malignancies 27,318 (64%; 20% allogeneic), solid tumors 1625 (4%; 2.9% allogeneic), and nonmalignant disorders 3063 (7%; 81% allogeneic). This year’s analysis focuses on cellular therapies with the marked growth in CAR T-cell therapies from 151 in 2017 to 301 patients reported in 2018. Other cellular therapy numbers show less significant changes. Important trends in HCT include a 49% increase in allogeneic HCT for chronic phase CML (although transplant numbers remain low) and a 24% increase in aplastic anemia. In autologous HCT, there is an ongoing increase in autoimmune diseases (by 19%), predominantly due to activity in multiple sclerosis. This annual report reflects current activity and highlights important trends, useful for health care planning.

## Introduction

Hematopoietic-cell transplantation (HCT) is an established procedure for many inherited or acquired disorders of the hematopoietic system, whether benign or neoplastic, including those of the immune system, and as enzyme replacement in metabolic disorders [1–3]. The activity survey of the European Society of Blood and Marrow Transplantation (EBMT), describing the status of HCT in Europe and affiliated countries, has become an instrument to observe trends and to monitor changes in the technology in Europe and neighboring countries [4–14]. The survey using a standardized structure captures the numbers of HCT from highly committed participating teams, divided by indication, donor type, and stem cell source. In the last years with the dramatic increase of cellular therapies, the survey also includes information on cellular therapies with hematopoietic cells for uses other than to replace the hematopoietic system [15–30]. The analysis of the survey data since 1990 shows a continued and constant increase in the annual numbers of HCT and transplant rates for both allogeneic and autologous HCT. This report, based on the 2018 survey data, shows recent trends, changes in indications, and use in Europe and the surrounding countries.

## Patients and methods

### Data collection and validation

Participating teams were invited to report their data for 2018 using the activity survey as listed in Table 1. The survey allows the possibility to report additional information on the numbers of subsequent transplants performed due to relapse, rejection, or those that are part of a planned sequential transplant protocol. Information on the numbers of patients receiving unmanipulated donor lymphocyte infusions (DLIs), nonmyeloablative, or reduced intensity HCT and the numbers of pediatric HCT is also collected.

**Table 1:** Numbers of HCT in Europe 2018 by indication, donor type and stem cell source.

	Transplant activity 2018																	
	No. of patients																	
	Allogeneic												Autologous			Total		
	Family									Unrelated								
	HLA-id			Twin	Haplo ≥ 2 MM		Other family						BM	BM+		Allo	Auto	Total
	BM	PBPC	Cord	All	BM	PBPC	BM	PBPC	Cord	BM	PBPC	Cord	Only	PBPC	Cord			
**Myeloid malignancies**	**312**	**2745**	**1**	**11**	**401**	**1102**	**6**	**79**	**1**	**461**	**5147**	**119**	**3**	**291**	**0**	**10,385**	**294**	**10,679**
Acute myeloid leukemia	220	1959	1	7	267	801	5	54	0	263	3279	87	3	290	0	6943	293	7236
1st complete remission	146	1280		5	135	399	5	34		175	1790	39	3	238		4008	241	4249
Not 1st complete remission	57	442	1	2	93	267		14		56	942	36		43		1910	43	1953
AML therapy related	7	68			8	32				7	162	2		1		286	1	287
AML from MDS/MPN	10	169			31	103		6		25	385	10		8		739	8	747
Chronic myeloid leukemia	12	107	0	0	13	34	0	1	0	21	176	8	0	0	0	372	0	372
Chronic phase	8	60			6	15		1		17	90	5				202	0	202
Not chronic phase	4	47			7	19				4	86	3				170	0	170
MDS or MD/MPN overlap	68	493		2	86	205	1	16		154	1275	22		1		2322	1	2323
MPN	12	186		2	35	62		8	1	23	417	2				748	0	748
**Lymphoid malignancies**	**350**	**1395**	**3**	**9**	**239**	**722**	**11**	**37**	**1**	**392**	**2135**	**75**	**37**	**21,912**	**0**	**5369**	**21,949**	**27,318**
Acute lymphatic leukemia	288	727	3	2	112	362	8	19	1	331	1046	66	4	70	0	2965	74	3039
1st complete remission	167	519	2	1	39	169	4	14		174	660	31	3	61		1780	64	1844
Not 1st complete remission	121	208	1	1	73	193	4	5	1	157	386	35	1	9		1185	10	1195
Chronic lymphocytic leukemia	6	39		1	1	20		1		7	116	1		10		192	10	202
Plasma cell disorders—MM	8	113		2	15	38		2		6	199	1	1	12,758		384	12,759	13,143
Plasma cell disorders—other		12			1	3					15	1		387		32	387	419
Hodgkin lymphoma	14	105		1	50	96	2	1		5	154	2	14	2107		430	2121	2551
Non Hodgkin lymphoma	34	399		3	60	203	1	14		43	605	4	18	6580		1366	6598	7964
**Solid tumors**	**5**	**3**	**0**	**0**	**4**	**25**	**0**	**0**	**0**	**3**	**7**	**0**	**32**	**1545**	**1**	**47**	**1578**	**1625**
Neuroblastoma	4	1			4	21					1		23	495		31	518	549
Soft tissue sarcoma/Ewing	1					2					1		4	241		4	245	249
Germinal tumors		2				1							1	380		3	381	384
Breast cancer										2				23		2	23	25
Other solid tumors						1				1	5		4	406	1	7	411	418
**Nonmalignant disorders**	**736**	**351**	**29**	**7**	**117**	**174**	**69**	**56**	**1**	**510**	**388**	**49**	**7**	**568**	**1**	**2487**	**576**	**3063**
Bone marrow failure—SAA	214	147	1	4	31	39	6	6		144	124	6		3	1	722	4	726
Bone marrow failure—other	68	21	3	1	15	13	4	10		64	31	2		1		232	1	233
Thalassemia	169	74	14	1	3	15	15	14		66	43			8		414	8	422
Sickle cell disease	110	46	8		12	13	9	4		17	6					225	0	225
Primary immune deficiencies	136	43	1	1	52	85	29	15	1	149	150	14	3	4		676	7	683
Inherited disorders of metabolism	37	13	2		3	8	5	6		64	33	27	3	3		198	6	204
Autoimmune disease	2	7			1	1	1	1		6	1		1	549		20	550	570
Others	38	29			12	16	4	14	1	27	45	9		21		195	21	216
**Total patients**	**1441**	**4523**	**33**	**27**	**773**	**2039**	**90**	**186**	**4**	**1393**	**7722**	**252**	**79**	**24,337**	**2**	**18,483**	**24,418**	**42,901**
Re/additional transplants	29	177	1	2	75	296	10	10		51	474	22	7	3413		1147	3420	4567
**Total transplants**	**1470**	**4700**	**34**	**29**	**848**	**2335**	**100**	**196**	**4**	**1444**	**8196**	**274**	**86**	**27,750**	**2**	**19,630**	**27,838**	**47,468**

In addition, centers can report information on specific transplants involving point of care and nonsubstantial processing of collected cells (such as immune cell selection) and on different types of cellular therapies that qualify as medicinal products since they result from substantial manipulations of the collected cells, whether industry and centrally manufactured or locally manufactured.

Quality control measures included several independent systems: confirmation of validity of the entered data by the reporting team, selective comparison of the survey data with MED-A data sets in the EBMT registry database and cross-checking with the National Registries.

### Teams

A total of 721 centers from 52 countries were contacted for the 2018 survey (43 European and 9 affiliated countries); of which 701 teams reported. This corresponds to a 97% return rate and includes 82% EBMT members and 18% non-EBMT members. Twenty active teams failed to report in 2018. Reporting teams are listed in the Supplementary online appendix in alphabetical order by country, city, and EBMT center code, with their reported numbers of first and total HCT, and of first allogeneic and autologous HCT as Supplementary material. The WHO regional office definitions were used to classify countries as European or non-European. Nine neighboring non-European countries participated in the 2018 EBMT survey: Algeria, Iran, Iraq, Israel, Jordan, Lebanon, Saudi Arabia, South Africa, and Tunisia. Their data, 3650 HCT in 3436 patients, from 38 actively transplanting teams make up 7.7% of the total data set and are included in all analyses.

### Patient and transplant numbers

Wherever appropriate, patient numbers corresponding to the number of patients receiving a first transplant in 2018, and transplant numbers reflecting the total number of transplants performed are listed. The term sibling donor includes HLA identical siblings and twins but not siblings with HLA mismatches. Unrelated donor transplants include HCT from matched or mismatched unrelated donors with peripheral blood and marrow as a stem cell source but not cord blood HCT. Haploidentical transplants are being described as any family member with two or more loci (but not more than five) mismatches within the loci HLA-A, -B, -C, -DRB1, and -DQB1 in GvH and/or HvG direction. Other family member donors are those related donors that are mismatched to a lesser degree than a full haplotype. Additional nonfirst transplants may include multiple transplants defined as subsequent transplants within a planned double or triple autologous or allogeneic transplant protocol, and retransplants (autologous or allogeneic) defined as unplanned HCT for rejection or relapse after a previous HCT.

### Hematopoietic cellular therapies other than hematopoietic-cell transplantation

Centers were requested to report all patients receiving hematopoietic cellular therapies in 2018. Hematopoietic cellular therapies were defined as infusion of cells, undergoing substantial manipulation after collection, either selection and/or expansion, or genetic modification and who thus qualify as investigational or approved ATMP’s according to Regulation (EC) N° 1394/2007. In this context, “substantial” should be understood as referring to the definition included in the Regulation and subsequent regulatory documents and may not reflect the workload assumed by cell-processing facilities working in conjunction with clinical programs. Depending on their nature and indications, hematopoietic cellular therapies may be designed to replace or to complement hematopoietic-cell transplants. Administration of nonsubstantially manipulated hematopoietic cells, such as transplantation of CD34+ selected hematopoietic stem cells is counted as HCT and not as cellular therapy [15]. Similarly, unmanipulated lymphocyte infusions post-HCT are counted as DLI and not as cellular therapy. Hematopoietic cellular therapies include what is defined in FACT-JACIE standards as immune effector cells; “A cell that has differentiated into a form capable of modulating or effecting a specific immune response” [16, 17]. This definition covers CAR-T cells and forms the basis for accreditation requirements in recent EBMT-JACIE recommendations [18].

Hematopoietic cellular therapies were categorized as chimeric antigen receptor T cells (CAR-T); in vitro selected and/or expanded T cells or cytokine activated, such as virus-specific T cells, cytokine-induced killer cells (CIK), regulatory T cells (TREGS), genetically modified T cells other than CAR-T, natural killer cells, dendritic cells, mesenchymal stromal cells, in vitro expanded CD34+ cells, and genetically modified CD34+ cells. This survey does not include cells from sources other than hematopoietic tissue [18–25]. On the other hand, gene therapy protocols, such as those used to treat thalassemia or SCID are part of this survey but numbers are currently very low.

### Transplant rates

Transplant rates, defined as the total number of HCT per 10 million inhabitants, were computed for each country without adjustments for patients who crossed borders and received their HCT in a foreign country. Population numbers for the European countries in 2018 were obtained from Eurostats: (http://appsso.eurostat.ec.europa.eu) and from the World Bank database for the non-European countries: (https://databank.worldbank.org).

Cellular therapies are shown on a map, as to where they are performed, but as numbers are still low, rates are not estimated.

### Analysis

Wherever appropriate, the absolute numbers of transplanted patients, transplants, or transplant rates are shown for specific countries, indications, or transplant techniques. Myeloid malignancy includes acute myeloid leukemia (AML), myelodysplastic or myelodysplastic/myeloproliferative neoplasia (MDS or MDS/MPN overlap), myeloproliferative neoplasm (MPN), and chronic myeloid leukemia (CML). Lymphoid malignancy includes acute lymphocytic leukemia (ALL), chronic lymphocytic leukemia (CLL), Hodgkin lymphoma, non Hodgkin lymphoma (NHL), and plasma cell disorders (PCD) (which includes multiple myeloma (MM) and others). The non malignant disorders include bone marrow failure (BMF) (which includes severe aplastic anemia (SAA) and other bone marrow failures), thalassemia and sickle cell disease (HG), primary immune disease (PID), inherited diseases of metabolism (IDM), and autoimmune disease (AID). Others include histiocytosis and other rare disorders not included in the above.

## Results

### Participating teams in 2018

Of the 701 teams, 456 (65%) performed both allogeneic and autologous transplants; 222 (32%) restricted their activity to autologous HCT, and 14 (2%) to allogeneic transplants only. Nine of the 701 responding teams (1%) reported no activity due to renovation or changes within the transplant unit. Within the 692 actively transplanting centers in 2018, 119 (17%) centers performed transplants on both adult and pediatric patients. An additional 122 (18%) centers were dedicated pediatric transplant centers and 451 (65%) centers perform transplants on adults only. Twenty teams failed to report in 2018, which, when compared with previously reported data, accounts for ~622 nonreported HCT.

### Numbers of patients, transplants, and trends

In 2018, 47,468 transplants were reported in 42,901 patients (first transplant); of these, 19,630 HCT (41%) were allogeneic and 27,838 (59%) autologous (Table 1). When compared with 2017, the total number of transplants increased by 4.5% (7.4% allogeneic HCT and 2.6% autologous HCT) [13]. The corresponding number of patients showed an increase of 7.7% for allogeneic HCT and 2.0% for autologous HCT. In addition, there were 4567 second or subsequent transplants, 1147 being allogeneic, mainly to treat relapse or graft failure and 3420 autologous, the majority of which were likely to be a part of multiple transplant procedures, such as tandem procedures, or as salvage autologous transplants for PCD. Furthermore, 1002 of the allogeneic HCTs were reported as being given after a previous autologous HCT, and were mainly for lymphoma or PCD.

The number of pediatric patients (under the age of 18) transplanted in both dedicated pediatric and joint adult–pediatric units was 5368 (4075 allogeneic and 1293 autologous). This is an overall increase of 6.2%. An increase was seen in allogeneic HCT of 9.4% and a decrease of 3% for autologous HCT when compared with 2017, where 5056 HCT (3725 allogeneic and 1331 autologous) were reported. Within the 5368 patients, 4207 transplants, and 3792 patients (2985 allogeneic; 79% and 807 autologous; 21%) were performed in dedicated pediatric centers. As seen in previous years, the increase in allogeneic HCT is most profound in pediatric patients (total allogeneic HCT 7.4% vs 9.4% in pediatric HCT). In total 22% of allogeneic HCT is performed in pediatric patients.

**Fig. 1:**
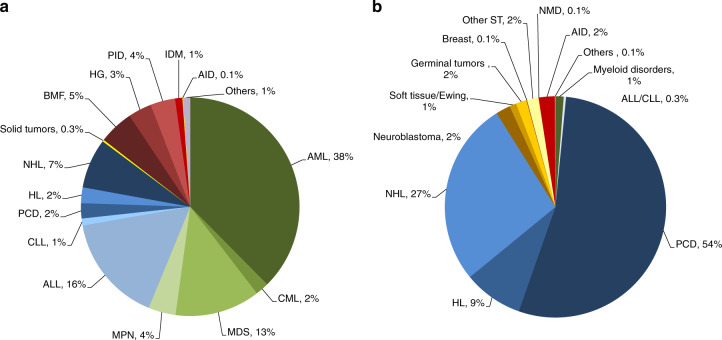
Relative proportion of disease indications for HCT in Europe 2018. **a** Relative proportion of allogeneic HCT. **b** Relative proportion of autologous HCT.

**Fig. 2:**
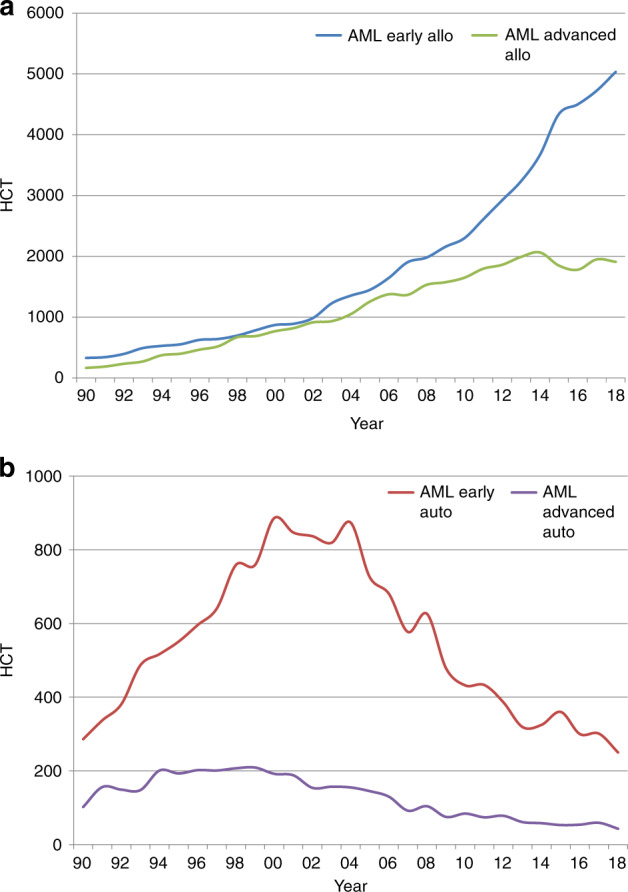
Changes in the use of HCT for AML. **a** Allogeneic HCT for AML in early and late disease. **b** Autologous HCT for AML in early and late disease.

Indications for HCT in 2018 are listed in detail in Table 1 (Fig. 1a, b). Main indications for HCT were myeloid malignancies (AML, CML, MDS or MDS/MPN overlap, and MPN): 10,679 (97% allogeneic HCT and 2.7% autologous HCT). The largest indication for allogeneic HCT is AML, 38% of all allogeneic HCT increasing by 4% when compared with 2017 [26]. In AML, only allogeneic HCT in early disease stage is increasing (Fig. 2a). Among the myeloid malignancies, CML has increased by 11% primarily in patients in chronic phase (48.5%) although overall the numbers remain low (*n* = 372 patients). Allogeneic HCT for MDS also continues to increase by 13% to 2322 patients treated. ALL comprises 16% of allogeneic HCT and showed an increase of 10.6% compared with the previous year [27]. Allogeneic HCT for CLL continues to decrease by 16.5%, a trend continued over the last years. Allogeneic HCT for NHL increased by 7.1% to 1366. Other important changes include the increase in allogeneic HCT for marrow failure, by 24% for SAA to 722 patients and by 6% to 232 patients for non-SAA marrow failure. Allogeneic HCT for primary immune deficiency increased by 22% to 676 and for inherited disorders of metabolism by 24.5% to 198 patients. In contrast, the number of allogeneic HCT for thalassemia and sickle cell disease appears to have stabilized after the increase in 2016 and 2017.

The main indications for autologous HCT are lymphoid malignancies (90%) with PCD comprising 54% of all autologous HCT patients. Autologous HCT for NHL has not changed over time and PCD has remained stable. A decrease was seen in autologous HCT for AML by 18.6% [28]. Overall, use of autologous HCT for AML has decreased over several years (Fig. 2b). A continued increase in autologous HCT for AID (19%) is observed, predominantly due to multiple sclerosis.

Figure 1a, b shows distribution of disease indications for allogeneic (Fig. 1a) and autologous (Fig. 1b) HCT as a pie graph.

**Fig. 3:**
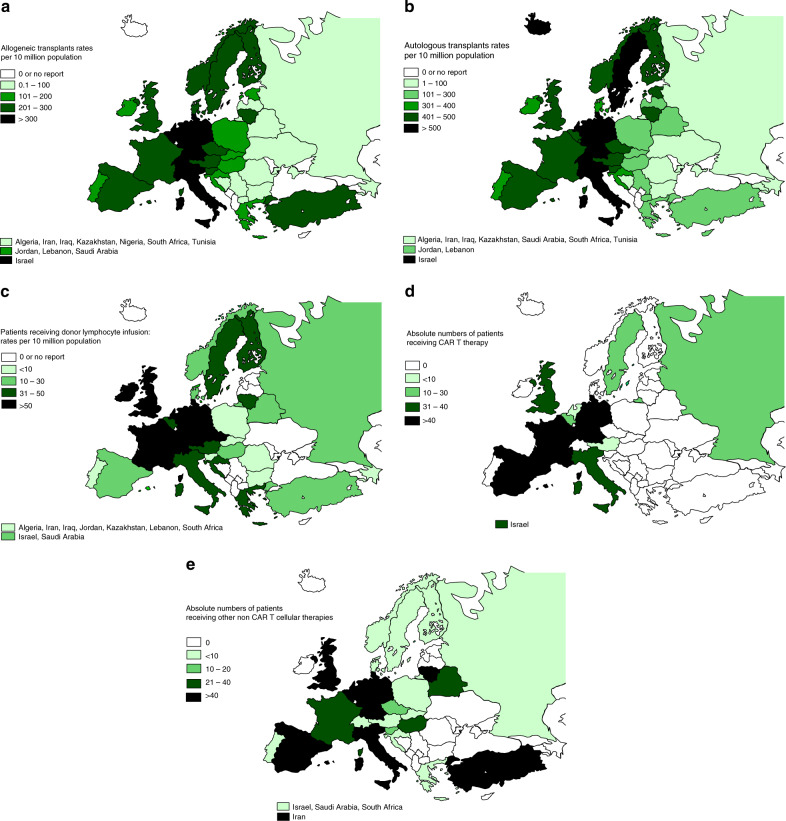
Transplant rates per 10 million population and absolute numbers in Europe 2018. **a** Transplant rates for allogeneic HCT. **b** Transplant rates for autologous HCT. **c** Rates for patients receiving donor lymphocyte infusions. **d** Absolute numbers of patients receiving CAR-T cellular therapy. **e** Absolute numbers of patients receiving other non-CAR-T cellular therapy.

Within allogeneic HCT 7392 were performed using nonmyeloablative or reduced intensity conditioning in 2018. This comprises 38% of all allogeneic HCT, and has remained stable over the last 10 years. European maps depicting transplant rates per 10 million population for allogeneic and autologous HCT are provided (Fig. 3a allogeneic HCT and 3b autologous HCT).

### Donor type and stem cell source

Since 2017, the numbers of family donors continues to rise with HLA identical sibling and syngeneic twin donors increased by 6% and haploidentical donors by 16.3% [13]. In sibling donor transplants, the use of peripheral blood stem cells increased by 6.7% and bone marrow stem cells by 3.5%. In haploidentical donor transplants, a higher increase is seen in the use of stem cells harvested from the bone marrow; 19%, while peripheral blood stem cells increased by 7.2% [10]. In unrelated donor transplants, the use of bone marrow and cord blood stem cells has decreased by 3.2% and 8.7%, respectively. However, after the slight decrease seen in 2016, the use of peripheral blood stem cells continues to increase again, 8% since 2017 [12]. Although the absolute numbers have increased in both allogeneic and autologous HCT, the proportion in use of bone marrow or peripheral blood stem cells remains stable throughout.

### Cellular therapy

Table 2 shows immune effector cell reinfusions, including DLI and cellular therapies performed in EBMT centers in 2018. There were 3096 patients receiving unmanipulated DLIs in 2018, an increase of 9.6% since 2017. The majority of DLI’s were given for relapse (1345) and graft enhancement (738). Figure 3c shows the rate of DLI use in European centers, per 10 million inhabitants, reflecting disparities in use of this technology by geographical region.

**Table 2:** Non-HCT cellular therapies using manipulated cells in 2018.

Number of patients	DLI	CAR-T		Selected/expanded T cells or CIK		Regulatory T cells (TREGS)		Genetically modified T cells		Natural killer cells		Dendritic cells		Mesenchymal stem cells		Genetically modified CD34+ cells		Other cell therapies		Total excluding DLI	
**2018**		Allo	Auto	Allo	Auto	Allo	Auto	Allo	Auto	Allo	Auto	Allo	Auto	Allo	Auto	Allo	Auto	Allo	Auto	Allo	Auto
GvHD				2		38						22		318		4		19		403	0
Graft enhancement	738			6						1		1		39		6		106	41	159	41
Autoimmune dis.													4	7	16					7	20
Genetic disease														2		9	6			11	6
Infection				97		1		5						10			1	4	2	117	3
Malignancy		19	282	15	2					12		21	29		10	1		24	2	92	325
DLI for residual disease	433																			0	0
DLI for relapse	1345																			0	0
DLI per protocol	580																			0	0
Regenerative medicine									1	1				25	33			10	71	36	105
**Total**	**3096**	**19**	**282**	**120**	**2**	**39**	**0**	**5**	**1**	**14**	**0**	**44**	**33**	**401**	**59**	**20**	**7**	**163**	**116**	**825**	**500**

Numbers of cellular therapies in Europe 2018 by indication, donor type and cell type.

A total of 1325 patients received other forms of hematopoietic cellular therapies that qualify as medicinal products rather than cell transplants [15]. The most widely used cellular therapy ahead of CAR-T cells in 2018 remains mesenchymal stromal cells (*n* = 460; 87% allogeneic), their use being mainly to treat graft-versus-host disease and expanded/selected T lymphocytes (*n* = 122; 98% allogeneic) [21]. However the most remarkable increase seen was in gene-modified T cells, notably CAR-T cells from 151 to 301 (100% increase) in patients treated in 2018. Most other cellular therapies appear to be decreasing slightly with exception of dendritic cells, which have increased from 44 in 2017 to 77 (75%) in 2018.

Figure 3d shows the absolute numbers of patients receiving CAR-T-cell therapies and Fig. 3e, other non-CAR-T-cell therapies by country.

## Discussion

The EBMT activity survey has been conducted annually since 1990 [7]. Over 47,000 transplants in almost 43,000 patients are reported in 2018. Allogeneic HCT appears now to expand more rapidly (7.1%) than autologous HCT (2.4%). In pediatric patients, the trend is even more pronounced, with an increase of 9.4% in allogeneic and a decrease of 3% in autologous HCT. This has changed from previous years where use of autologous HCT has been expanding more rapidly. In previous years we had observed a slower growth for unrelated donor HCT as compared for haploidentical HCT [12]. In the last year, however, use of both types of donors appears to increase simultaneously; 16% increase for haploidentical donors, 5% for unrelated donors, but similar increases in absolute numbers. Indications have not changed dramatically. It is mostly in well-established indications where growth is observed, such as allogeneic HCT for AML in CR1 but also ALL, more so with advanced disease than with CR1 patients [26, 27]. This might reflect the use of new treatments in ALL, such as bispecific antibodies or drug-conjugated antibodies, allowing relapsed patients access to transplant. Use of allogeneic HCT in CLL continues to drop reflecting the availability of innovative-targeted drugs.

Last year, we reported lower rates of aplastic anemia transplantation possibly due to the use of thrombopoietin analogs such as eltrombopag. In 2018, however, we see the number has increased by 24% compared with 2017, possibly suggesting that transplants are now performed later after failing thrombopoietin analogs. The more frequently use of alternative donors, such as haploidentical HCT and the more accepted indications for inherited disorders might also explain those results. Interpretation of these trends is obviously preliminary, as we do not have data on the use of treatment alternatives.

The most impressive growth is observed in hematopoietic cellular therapies, most notably in the use of CAR-T cells, increasing to 301 reported patients treated in 2018. Since the only two approved products received a centralized marketing approval from EMA in August 2018, it is likely that the reported activity for 2018 mostly and partially reflects clinical studies, either industry-sponsored or academia-sponsored (https://www.ema.europa.eu/en/documents/scientific-guideline/qualification-opinion-cellular-therapy-module-european-society-blood-marrow-transplantation-ebmt_en.pdf). Investigational CAR-T cells can be produced by academic facilities in the context of the hospital exemption, a specific provision embedded in the Regulation (EC) 1394/2007. Emergence of a rapidly growing clinical activity is reassuring in view of earlier reports demonstrating that Europe lagged behind the USA and China [29]. Autologous or allogeneic HCT for NHL has not changed over time and use of the transplant technology for PCD has remained stable. These are indications for autologous as well as for allogeneic HCT which may in the future be replaced by CAR-T treatments. Since the EMA approved the first CAR-T product, a notable increase in the use of CAR-T cells has been observed and a further increase is to be expected in 2019. Hematopoietic cellular therapies other than CAR-T are mostly decreasing. This may be due to centers focussing more on developing CAR-T treatment protocols, or because academic facilities that were historically involved in the development of these innovative treatments face ever more stringent conditions for manufacturing, in relation with the regulatory framework and the advent of industry-manufactured somatic cell therapy medicinal products or gene therapy medicinal products. In addition, our data may reflect a certain amount of underreporting as patients treated on trials for CAR-T cells may not be reported in the survey due to trial regulations. The inclusion of a Cellular Therapy Form to the EBMT registry was a key determinant in EBMT receiving a positive opinion from EMA in February 2019. This will help EBMT, together with all interested parties, to contribute to PASS and PAES studies, that are much needed to establish the true medical value of these costly treatments and more accurately define the safety profile of these gene therapy medicinal products for which both the FDA and EMA mandated a 15 year follow-up. The future of CAR-T therapy is obviously open at this point in time given successes [24] but also risks of failure [25].

EBMT centers will continue the well-established practice of transparently sharing data on activity of advanced therapy medicinal products manufactured from hematopoietic cells used and on the outcome of patients. Developing high-quality data reporting tools for novel cellular therapies will add to the established track record of EBMT organizing HCT teams to maximize the availability of data across the rapidly advancing and expanding field of HCT and cellular therapy. The annual activity survey of the EBMT reflects current activity and trends in the field of transplant technology. It is valuable for the dissemination of the most recent information on indications, donor and stem cell usage, and benchmarking of data completeness, and survival outcomes [30], which can ultimately be beneficial in health care planning.

## Supplementary information

Appendix of reporting centers
